# Risk factors, management and outcomes for peritoneal dialysis access damage

**DOI:** 10.1080/0886022X.2024.2425161

**Published:** 2024-11-11

**Authors:** Ruoxi Liao, Xueli Zhou, Xia Liu, Xueqin He, Li Pu, Dengyan Ma, Hui Zhong

**Affiliations:** Department of Nephrology, West China Hospital, Sichuan University, Chengdu, Sichuan Province, China

**Keywords:** Peritoneal dialysis, peritoneal dialysis catheter, risk factor, management, outcome

## Abstract

**Objectives:**

Peritoneal dialysis (PD) access damage is an uncommon complication of PD. This study aimed to describe the characteristics, management and outcomes of PD access damage.

**Methods:**

This retrospective study included patients who suffered from PD access damage between January 2018 and January 2024 at the PD Center of West China Hospital. Patient characteristics and access damage information were collected from medical records.

**Results:**

A total of 128 PD patients without PD access damage were included in the control group. A total of 45 patients (51% male; aged 58 ± 14 years) suffered from 49 cases of PD access damage. Multivariate logistic analysis revealed that previous peritonitis [odds ratio (OR) 3.93; 95% confidence interval (CI) 1.56 to 9.94] and assisted PD (OR 4.20; 95% CI 1.25 to 14.12) were risk factors, while catheter belt use (OR 0.16; 95% CI 0.06 to 0.44) and training frequency per year (0.34; 95% CI 0.19 to 0.64) were protective factors against PD access damage. Managements included cutting and repairing (*N* = 24), transfer set replacement (*N* = 11), catheter removal (*N* = 9) and catheter replacement (*N* = 4). Thirty-nine patients continued with PD after access repair and were followed up for a median of 35 months. The repaired access functioned well during follow-up.

**Conclusions:**

In conclusion, to avoid PD access damage, catheter belt and routine retraining are recommended. Once PD access damage is identified, patients should clamp the catheter, take prophylactic antibiotics and resort to the treatment team. The treatment team should evaluate PD access and handle it according to a clinical protocol.

## Introduction

Peritoneal dialysis (PD) is an established renal replacement therapy for end-stage kidney disease (ESKD) that offers patients more flexibility and independence than does hemodialysis. Functional PD access is crucial for performing high-quality PD. PD access typically comprises three parts: an external catheter, a catheter adaptor and a transfer set. Mechanical complications, including access damage, can lead to catheter loss and contribute to technique failure [1, 2].

PD access damage may result from artificial injuries [3], such as cuts, punctures, chemical applications and manufacturing errors, and can also present as spontaneous ruptures [4]. This complication is rarely noted in the literature. Some case reports have described patients with spontaneous catheter ruptures or ruptures caused by external forces [4–10]. According to one case report, spontaneous catheter rupture may be related to mupirocin application [7]. However, the risk factors for PD access damage remain unclear.

The Internal Society for Peritoneal Dialysis (ISPD) suggested several management strategies for external catheter damage, including external splicing repair, internal splicing repair and catheter replacement [1]. As catheter damage with leakage is considered a contaminating event, prophylactic antibiotics are suggested [1]; however, there are no clinical trials to directly support this treatment. Some PD centers perform peritoneal washing after catheter repair or replacement, but whether the washing procedure could benefit the patient is uncertain. Nowadays, there is a lack of standard management protocols for PD access damage.

The aim of this study was to describe the risk factors, characteristics and outcomes of patients with PD access damage and to discuss the management of these patients following PD access damage.

## Methods

In this retrospective study, PD patients who suffered from PD access damage at the PD Center, West China Hospital, between January 2018 and January 2024 were analyzed. The inclusion criteria were as follows: (1) patients on maintenance peritoneal dialysis and (2) patients who suffered from PD access damage between January 2018 and January 2024. We also included 128 age- and sex-matched PD patients without PD access damage at our PD center between January 2018 and January 2024 as the control group. Patients were excluded if they damaged the PD access on purpose. The study was approved by the Ethics Committee of the West China Medical Center of Sichuan (No. 2019-793). Informed consent was obtained from all the patients during follow-up. The study was carried out in accordance with the principles of the Declaration of Helsinki and reported according to the STROBE guidelines.

### Data collection

The PD center keeps electronic records, including demographic characteristics, complications, PD catheterization data and outcomes (peritonitis, transfer to hemodialysis, renal transplantation and death), for all PD patients. When a patient suffered from PD access damage, damage-related information, including damage location, size, management, previous chemical exposure, catheter immobilization status and catheter flexion, was routinely recorded. Chemical exposure was defined as the application of disinfectant or antibiotics to the external catheter for more than once per month. The catheter position at an acute angle was regarded as catheter flexion. Baseline information and PD catheter damage data were extracted from those records. Information about usual catheter care and catheter position for the control group was collected *via* face-to-face interviews during routine PD follow-up.

### PD access damage

For all the patients, a 41-cm Tenckhoff 2 cuff straight PD catheter (Argyle, Covidien), a titanium adaptor (Baxter) and a MiniCap extended life PD transfer set with a twist clamp (Baxter) were used. The transfer set was routinely replaced every 6 months. PD access damage was defined as damage at any part of the PD access that results in PD access defects or dysfunction. The PD access was evaluated by a PD nurse, and PD access damage was diagnosed if leaks or breaks in either the PD catheter, adaptor or transfer set were noticed.

### PD access damage management

**Figure 1.**
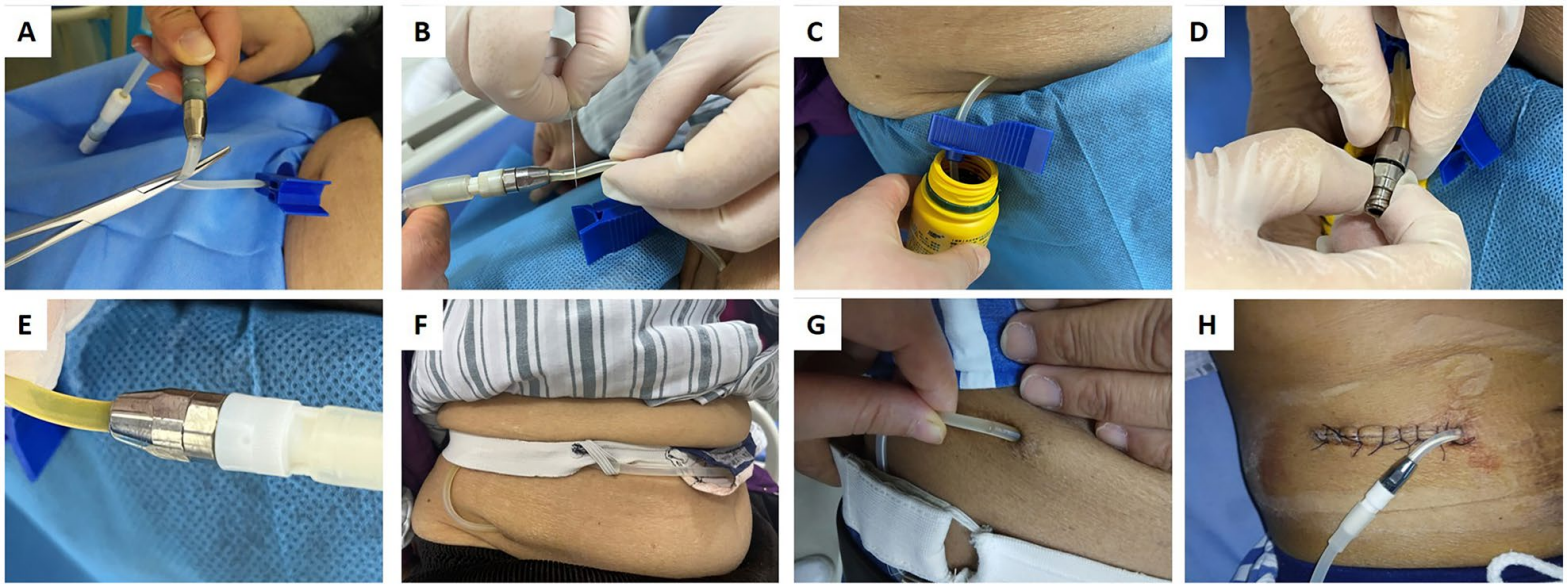
Catheter management after damage. (A–F) For damage more than 5-6 cm away from the exit site, the catheter was cut proximally to the breakage site with the proximal part clamped (A, B), the remaining part of the external catheter was soaked in iodophor for 15 min (C) and connected to the adapter (D, E), and the repaired catheter was immobilized with a belt (F). (G, H) For damage less than 5–6 cm away from the exit site (G), an incision was made to expose the catheter, the breakage region was cut, and the adapter was connected to the remaining part of the subcutaneous catheter (H). In this case, as the distance between the breakage site and the outer cuff was too short, the catheter was cut proximally to the outer cuff.

We handled external catheter damage according to the distance between the breakage site and the exit site. If the breakage site was more than 5–6 cm away from the exit site, the breakage part was cut, and the adaptor was connected to the remaining part of the external catheter (Figure 1(A–F)). If the breakage site was within 5–6 cm from the exit site and no peritonitis was identified, we considered simultaneous PD catheter removal and reinsertion. If the breakage site was within 5–6 cm away from the exit site without peritonitis and the patient strongly preferred catheter repair, a 2- to 5-cm incision was made to expose the catheter, the breakage site (with or without the outer cuff) was cut, and the adapter was connected to the remaining part of the catheter (Figure 1(G,H)). If the distance between the breakage site and exit site was less than 5–6 cm with concomitant peritonitis or if the patient chose to transfer to hemodialysis, the catheter was removed. For patients with transfer set damage, transfer set replacement was performed.

### Outcomes

All patients were followed up until death or January 15, 2024. Follow-up was performed by checking medical records, follow-up visits and telephone interviews. The outcomes included peritonitis, transfer to hemodialysis, renal transplantation and death. Peritonitis was defined according to the International Society for Peritoneal Dialysis (ISPD) 2022 peritonitis guidelines [11].

### Statistical analysis

Categorical variables were expressed as numbers with percentages. Continuous variables were expressed as the mean with standard deviation or median with interquartile range (IQR). The chi-square test was performed to compare proportions, and the two-sample t test or Wilcoxon rank sum test was performed to compare continuous variables. Risk factors for PD access damage were identified with multivariate logistic regression, with a P value of 0.15 for the backward stepwise procedure. The eligible candidate variables included age, sex, height, dialysis duration, impaired vision, previous peritonitis history, urine volume, Kt/V, dialysate volume, residual glomerular filtration rate (GFR), assisted PD, catheter belt use, catheter flexion and training frequency. The level of significance was set at 0.05. All analyses were performed using Stata 11.0 (StataCorp. 2011, TX: StataCorp LP).

## Results

### Patients

A total of 49 cases of PD access damage were identified among 45 patients (51% male; aged 58 ± 14 years) between January 2018 and January 2024. The PD access damage rate in our PD center was 0.028 episodes/patient-year. All the patients with PD access damage were on continuous ambulatory peritoneal dialysis (CAPD) with a median dialysis duration of 97 months (IQR 52 to 126 months). We also included 128 age- and sex-matched PD patients without PD access damage at our PD center as control. All the patients in both groups used the same PD catheter without catheter exchange or embedding from the commencement of PD. The characteristics of the patients with and without PD access damage are presented in Table 1.

**Table 1.** Characteristics of patients with and without PD access damage.

		Without damage	PD access damage	*P*
N		128	45	
Age, years		54 ± 14	58 ± 14	0.28
Dialysis duration, month		35 (29-88)	97 (52-126)	<0.001
Height, cm		160 ± 10	162 ± 8	0.08
Weight, kg		57 ± 11	59 ± 13	0.44
Male		56 (44%)	23 (51%)	0.39
Complication	Diabetes	27 (21%)	7 (16%)	0.42
	Hypertension	117 (91%)	39 (87%)	0.36
	CVD	25 (20%)	14 (31%)	0.11
	Impaired vision	46 (36%)	25 (56%)	0.02
Marriage	Married	109 (85%)	36 (80%)	0.32
	Unmarried	12 (9%)	8 (18%)	
	Divorced	4 (3%)	0	
	Widowed	3 (2%)	1 (2%)	
Education	Illiteracy	3 (2%)	1 (2%)	0.81
	Primary school	23 (18%)	4 (9%)	
	Middle school	40 (31%)	16 (37%)	
	High school	31 (24%)	11 (26%)	
	University	30 (23%)	11 (26%)	
	Postgraduate	1 (1%)	0	
Assisted PD		9 (7%)	8 (18%)	0.04
Chemical exposure	H_2_O_2_	0	1 (2%)	0.09
	Gentamicin	0	0	
	Mupirocin	60 (47%)	26 (58%)	0.21
	Lodophor	118 (92%)	44 (98%)	0.19
Catheter belt use		64 (50%)	10 (22%)	0.001
Catheter flexion		57 (45%)	34 (76%)	<0.001
Lap catheter implantation		33 (26%)	9 (20%)	0.61
Training, /year		2.5 ± 0.8	1.7 ± 0.9	<0.001
PD exchange, /day		4 (3-4)	4	0.37
Dialysate volume, l/day		7.4 ± 1.7	7.5 ± 1.9	0.37
Urine volume, ml/day		125 (0-600)	0 (0-100)	0.001
Kt/V, /w		1.93 ± 0.48	1.86 ± 0.35	0.37
GFR, ml/min		2.7 (0-20.2)	0 (0-2.3)	0.002
Icodextrin		15 (12%)	3 (7%)	0.34
Previous peritonitis		30 (23%)	22 (49%)	0.001

Continuous variables are presented as the mean ± standard deviation or median (interquartile range). Abbreviations: CVD, cerebral vascular disease; Lap, laparoscopic; H_2_O_2_, hydrogen peroxide; GFR, glomerular filtration rate.

### Risk factors for PD access damage

Compared with patients without PD access damage, patients with PD access damage had higher rates of impaired vision and previous peritonitis, longer PD duration, less urine volume, lower training frequency, more catheter flexion, less catheter belt use and more assisted PD. Further multivariate logistic analysis showed that previous peritonitis [odds ratio (OR) 3.93; 95% confidence interval (CI) 1.56 to 9.94] and assisted PD (OR 4.20; 95% CI 1.25 to 14.12) were risk factors, while catheter belt use (OR 0.16; 95% CI 0.06 to 0.44) and training frequency per year (0.34; 95% CI 0.19 to 0.64) were protective factors for PD access damage (Supplementary Table 1). All the assisted PD were provided by family members. Catheter flexion was noted in 32 patients with a lack of belt use, 21 patients with inconvenient exit site locations and 13 patients with both. Catheter flexion was a risk factor for PD access damage in univariate analysis but not in multi-variate analysis. All four patients who suffered from PD access damage twice had PD catheter flexion with a lack of belt use.

### Characteristics of PD access damage

**Figure 2.**
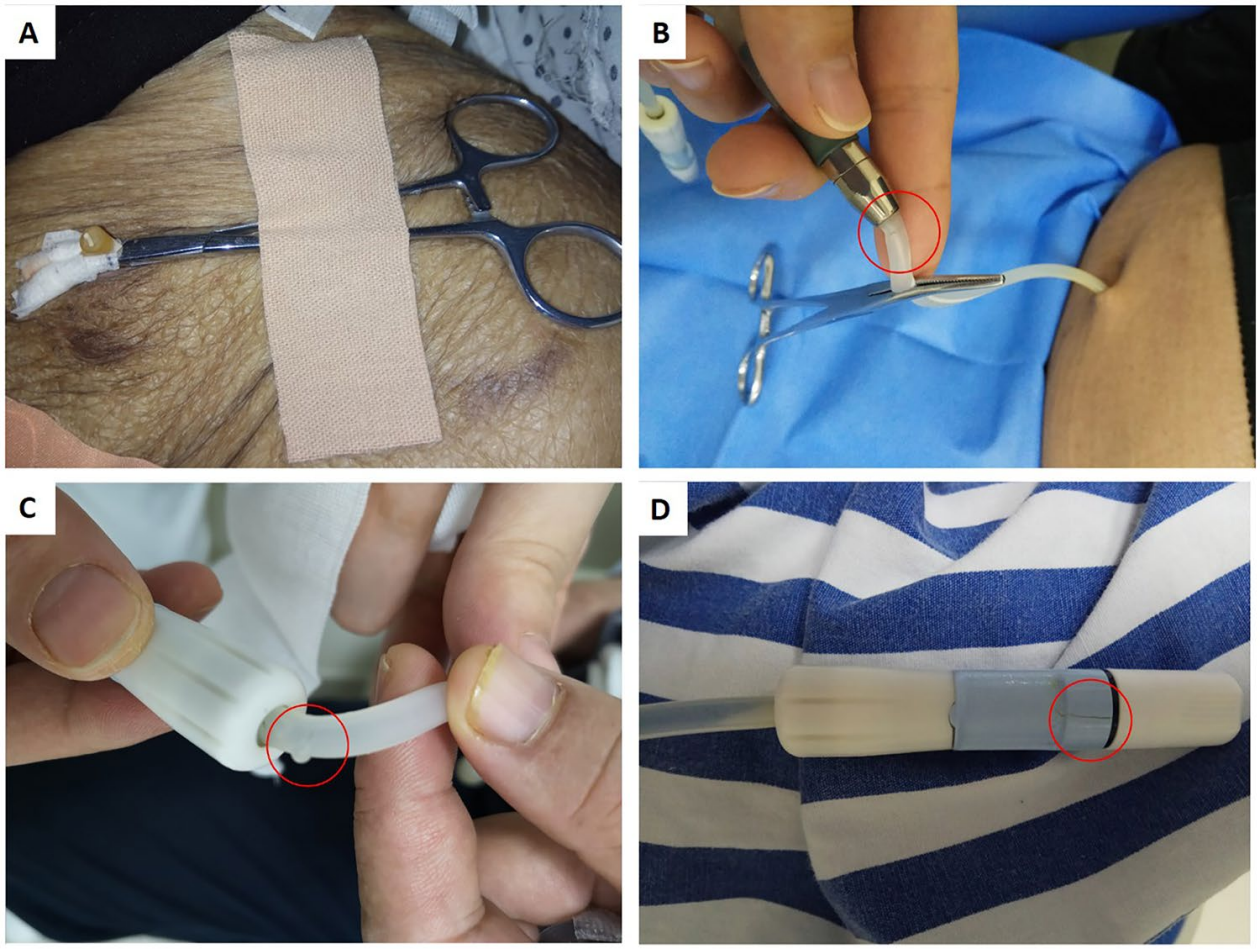
PD access damage. (A) Cut damage close to the exit site. (B) A tubing laceration at the external catheter close to the catheter adaptor. (C) Transfer set catheter damage. (D) Transfer set twist clamp crack (the red circle shows the breakage.).

Figure 2 shows PD access damage at different locations. The damage was caused by sharp instrument cuts or punctures (*n* = 9, 18%), catheter adapter tears (*n* = 3, 6%), chemical destruction (*n* = 2, 4%) or no obvious cause (*n* = 35, 71%). A total of 38 injuries occurred at the external catheter, and 11 occurred at the transfer set. Most of the injuries were located near the exit site, the adaptor and the transfer set clamp (Supplementary Figure 1).

### PD access damage management

In total, 24 patients underwent catheter cutting and repair, 4 patients underwent catheter replacement, 9 patients underwent catheter removal, and 11 patients underwent transfer set replacement. Seven of the patients who underwent catheter removal were on both hemodialysis and peritoneal dialysis before PD access damage, and catheter removal was based on patient preference. Thirty-two patients underwent peritoneal washing after the procedure. Forty patients received anti-infective treatment before visiting the hospital, while 9 patients did not. One patient died soon after admission with the catheter clamped, and no further management was performed.

### Peritonitis at the time of access damage

At the time of access damage, 8 patients (16%) were diagnosed with peritonitis (6 with gram-positive bacterial peritonitis and 2 with culture-negative peritonitis). Among the patients with peritonitis, 6 patients had external catheter damage, and 2 patients had transfer set catheter damage. Compared with patients without peritonitis, a greater proportion of patients with peritonitis did not clamp the catheter proximally to the breakage site before going to the hospital (20% vs. 61%, *p* = 0.01).

### Patient outcomes during follow-up

**Figure 3.**
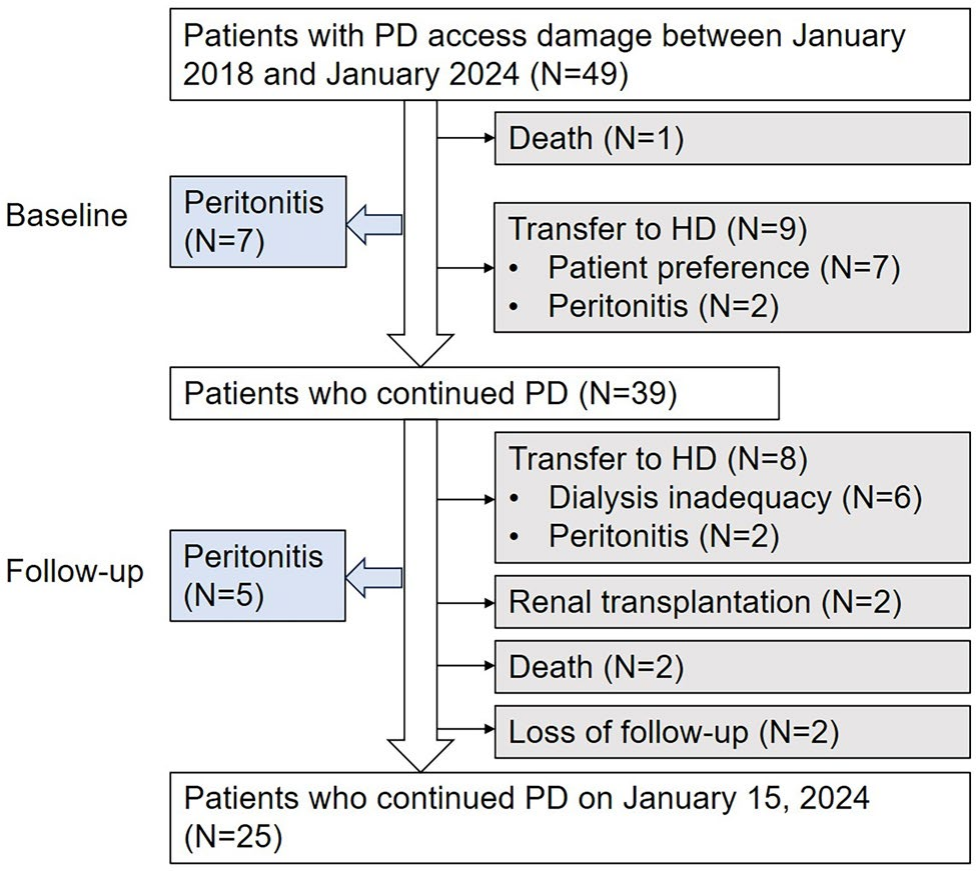
Patient outcomes with PD access damage. Abbreviations: HD, hemodialysis.

Patient outcomes are presented in Figure 3. Thirty-nine patients continued with PD after access repair and were followed up for a median of 35 months (IQR 13 to 50 months).

The repaired access functioned well during follow-up. No new-onset/recurrent/relapsing peritonitis was identified within 4 weeks following catheter repair or the completion of anti-infective therapy. During the long-term follow-up, 5 patients suffered from peritonitis, which all resulted from Staphylococcus infection. No cases of repeat peritonitis were identified.

A total of 17 patients were transferred to hemodialysis, and 3 patients died. One patient was admitted to the hospital 4 days after the external catheter was cut close to the exit site and died from respiratory failure soon after admission. The other 2 patients died more than one year after PD access damage.

## Discussion

This study described the characteristics and outcomes of 45 patients with 49 cases of PD access damage. PD access damage was associated with previous peritonitis, catheter belt use and training frequency. Clamping the catheter when PD access damage was identified helped lower the risk of peritonitis.

Several risk factors for PD access damage were identified in this study. Some studies have suggested that mupirocin may result in structural changes in silicon rubber PD catheters [12] and may injure polyurethane Cruz catheters [7]. Our study did not find any relationship between chemical exposure and access damage. On the other hand, we found that previous peritonitis was an independent risk factor for access damage. It is suggested that macrophages may damage the polyurethane polymer [13, 14]. Whether microorganisms and human cells can degrade silicon catheters requires further study.

Univariate analysis revealed that catheter flexion was also a risk factor for PD access damage. PD access damage was mostly distributed close to the exit site, the adaptor or the clamp, corresponding to sites vulnerable to flexing. Although a previous study which simulated daily routine usage of PD catheters did not detect any material defects under physical stress [3], it is possible that recurrent flexion may lead to crazes, which grow gradually and lead to breakage or change the microstructure inside the catheter and reduce catheter resistance. Our study also revealed that catheter belt use and retraining were protective factors against access damage. So, catheter flexion should be avoided, while catheter belt use and routine retraining (e.g. 3 times/year) are recommended.

Most patients with external catheter damage were treated with catheter cutting and repair in our study. According to our study, cutting and repair can save broken catheters without interrupting the PD, with good early and long-term outcomes. This procedure requires no extra materials, making it easy for PD centers to perform. According to our clinical practice, a distance of no less than 5–6 cm from the exit site is required to perform cutting and repair for the convenience of future exit site care and PD treatment. If the distance is not long enough, catheter exchange or catheter repair with skin cutting may be considered. Some previous studies suggested other treatments, e.g. with a repair kit [15], two PD adaptors [16] or manipulated material [17], which can also be considered.

**Figure 4.**
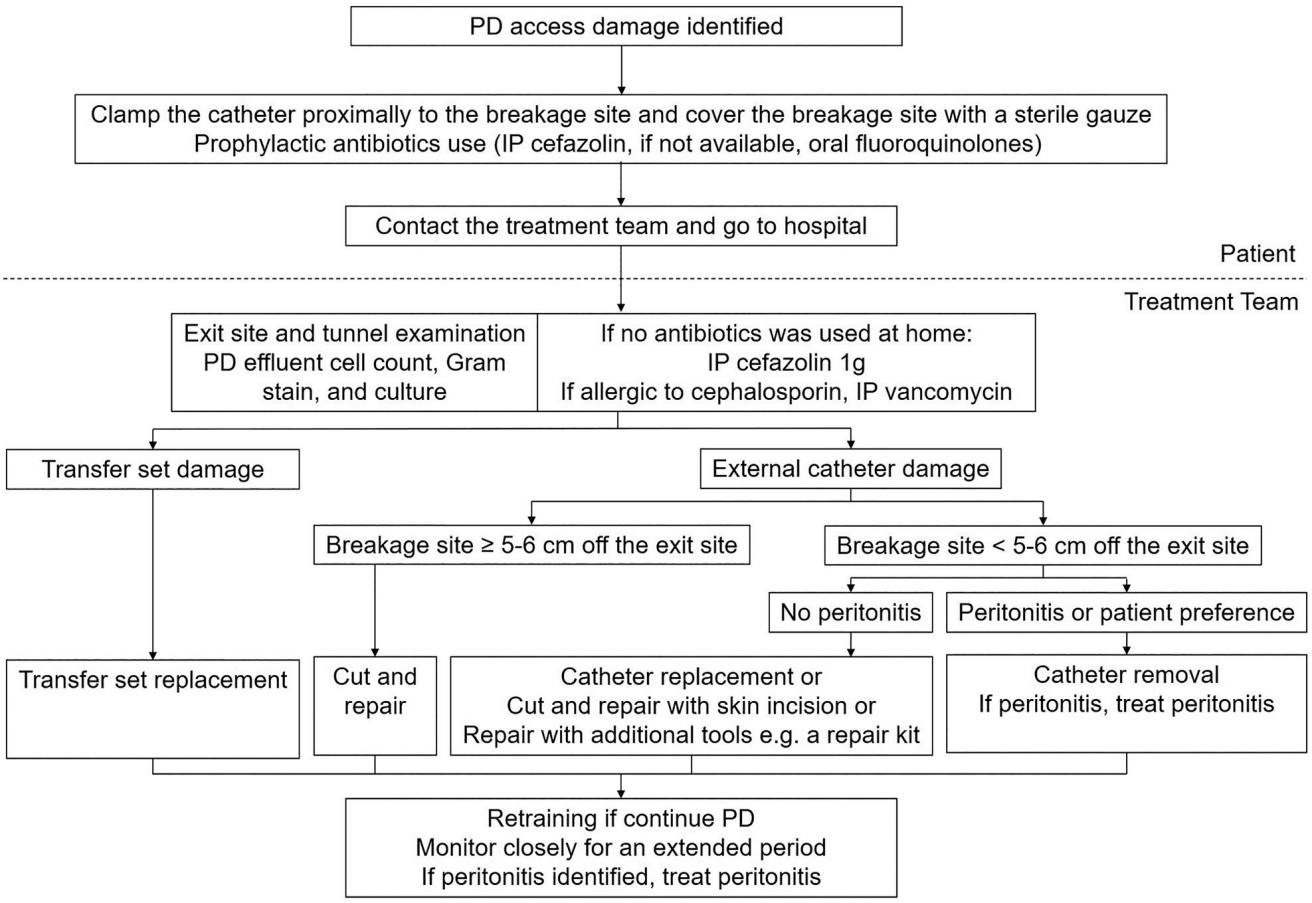
A protocol for treatment of PD access damage. Abbreviations: IP, intraperitoneal.

The rate of peritonitis after PD access damage in our study was 16%. Damage to the PD catheter can result in wet contamination of the PD system, which refers to contamination with an open system, when either dialysis fluid is infused after contamination or if the catheter administration set has been left open for an extended period [11]. According to a retrospective study, the incidence of peritonitis after wet contamination was 5.63% and prophylactic antibiotics significantly reduced the risk of peritonitis [18]. Thus, ISPD suggests prophylactic antibiotics after wet contamination [11]. Our study showed that the risk of peritonitis was decreased by clamping the catheter before the patient went to the hospital. The benefits of postprocedure peritoneal washing and prophylactic antibiotics were not confirmed. However, considering the high rates of peritonitis in patients with PD access damage, antibiotics are recommended. Due to a lack of clinical evidence, there is no standard regimen of prophylactic antibiotics, one dose of intraperitoneal cefazolin was suggested by ISPD guideline [11]. Therefore, we recommend that once PD access damage is identified, the patient should clamp the proximal part of the catheter, take prophylactic antibiotics and go to the hospital. A proposed protocol after PD access damage was listed in Figure 4.

This study has several limitations. First, as PD access damage is an uncommon complication, the sample size of this study was small, which may reduce the power of the study, and some risk factors for PD access damage may not be identified. Perhaps due to the small sample size, we didn’t find a difference in peritonitis rate between patients with and without anti-infective treatment, which needs further larger studies. Second, we proposed a protocol for treatment of PD access damage depending on our clinical practice. There is insufficient evidence to support some of the treatments, e.g. the dose and duration of prophylactic antibiotics and the distance cutoff values between the breakage site and exit site which determine catheter management choice, and need further study. Third, as a retrospective study, we did not look into the structure of the broken catheter, e.g. with light microscopy or scanning electron microscopy. Further studies on catheter ultramicrostructure may be performed to explore the mechanisms of PD access damage. Forth, due to the limited number of PD patients in our center and relatively long PD duration in the PD access damage cohort, we didn’t match the control group by dialysis vintage, which may impact the accuracy of our estimates of the risk factors for PD access damage.

## Conclusion

In conclusion, our study described the characteristics and outcomes of PD patients with PD access damage and proposed a protocol after PD access damage. PD access damage was associated with previous peritonitis, catheter belt use, assisted PD and training frequency. Clamping the catheter when PD access damage was identified helped lower the risk of peritonitis.

## Supplementary Material

supplementary fig 1.jpg

## Data Availability

All data generated or analyzed during this study are included in this article. Further enquiries can be directed to the corresponding author.
