# Supplementary figures and images for: Risk factors, management and outcomes for peritoneal dialysis access damage
Source: Ren Fail. 2024 Nov 11;46(2):2425161. doi: 10.1080/0886022X.2024.2425161 (PMC11556276; doi:10.1080/0886022X.2024.2425161)

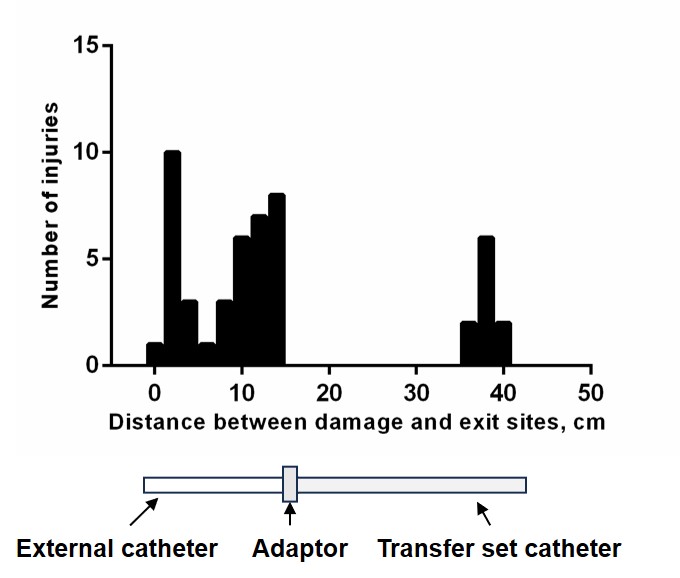

Supplement: supplementary fig 1.jpg [file IRNF_A_2425161_SM1911.jpg]
